# Exploring the Biological Activity of a Humanized Anti-CD99 ScFv and Antibody for Targeting T Cell Malignancies

**DOI:** 10.3390/biom14111422

**Published:** 2024-11-08

**Authors:** Nuchjira Takheaw, Thanathat Pamonsupornwichit, Ratthakorn Chaiwut, Kamonporn Kotemul, Kanokporn Sornsuwan, On-anong Juntit, Umpa Yasamut, Passaworn Cheyasawan, Witida Laopajon, Watchara Kasinrerk, Chatchai Tayapiwatana

**Affiliations:** 1 Division of Clinical Immunology, Department of Medical Technology, Faculty of Associated Medical Sciences, Chiang Mai University, Chiang Mai 50200, Thailand; nuchjira.t@cmu.ac.th (N.T.); kote_kamonp@hotmail.com (K.K.); umpa.yas@cmu.ac.th (U.Y.); witida.l@cmu.ac.th (W.L.); 2 Biomedical Technology Research Center, National Center for Genetic Engineering and Biotechnology, National Science and Technology Development Agency, Faculty of Associated Medical Sciences, Chiang Mai University, Chiang Mai 50200, Thailand; ratthakorn_c@hotmail.com; 3 Center of Biomolecular Therapy and Diagnostic, Faculty of Associated Medical Sciences, Chiang Mai University, Chiang Mai 50200, Thailand; thanathat.pamon@cmu.ac.th (T.P.); kanokporn.sornsuwan@cmu.ac.th (K.S.); onanong.j@cmu.ac.th (O.-a.J.); 4 Office of Research Administration, Chiang Mai University, Chiang Mai 50200, Thailand; 5 Department of Pathology, Faculty of Medicine Ramathibodi Hospital, Mahidol University, Bangkok 73170, Thailand; passaworn.cheyasawan@gmail.com

**Keywords:** CD99, T cell malignancies, antibody humanization, antibody therapy, apoptosis

## Abstract

CD99, a type I transmembrane protein, emerges as a promising therapeutic target due to its heightened expression in T cell acute lymphoblastic leukemia (T-ALL). This characteristic renders it a potential marker for minimal residual disease detection and an appealing target for antibody-based treatments. Previous studies have revealed that a mouse monoclonal antibody, mAb MT99/3, selectively binds to CD99, triggering apoptosis in T-ALL/T-LBL cells while preserving the integrity of healthy cells. By targeting CD99, mAb MT99/3 suppresses antigen presentation and disrupts T cell functions, offering promise for addressing hyperresponsive T cell conditions. To facilitate clinical translation, we developed a humanized ScFv variant of mAb MT99/3, termed HuScFvMT99/3 in “ScFvkh” design. Structural analysis confirms its resemblance to the original antibody, and the immunoreactivity of HuScFvMT99/3 against CD99 is preserved. The fully humanized version of antibody HuMT99/3 was further engineered, exhibiting similar binding affinity at the 10^−10^ M level and specificity to the CD99 epitope without antigenic shift. HuMT99/3 demonstrates remarkable selectivity, recognizing both malignant and normal T cells but inducing apoptosis only in T-ALL/T-LBL cells, highlighting its potential for safe and targeted therapy.

## 1. Introduction

Antibody therapies play a pivotal role in treating a wide range of diseases, including cancer, autoimmune disorders, and infectious diseases [1,2]. As of 30 June 2022, a total of 162 monoclonal antibodies (mAbs) have received approval for antibody therapy. Their efficacy and safety are attributed to properties such as high specificity and diverse mechanisms of action, making them valuable therapeutic agents [1,3]. These antibody drugs hold substantial market value, estimated at around USD 115.2 billion in 2018 on a global scale. Moreover, the antibody drug market is poised for significant growth, with projections indicating it could reach USD 300 billion by 2025 [3]. Despite progress, developing new, more diverse antibodies will offer patients access to a wider range of treatment options and potentially more successful outcomes.

T lymphoblastic leukemia/lymphoma is a heterogeneous group of disorders, including T acute lymphoblastic leukemia (T-ALL) and T lymphoblastic lymphoma (T-LBL). T lymphoblastic leukemia/lymphoma is an aggressive hematologic malignancy characterized by clonal growth of immature T cell progenitors [4,5]. Currently, the frontline treatment is intensified multiagent chemotherapy frequently causing several adverse effects with insufficient efficacy [6,7]. Therefore, there is a need for the development of novel non-chemotherapeutic strategies for T-ALL/T-LBL, particularly antibody therapy. Currently, there is a limited development of antibody drugs for treating T cell malignancies. A significant challenge is that many tumor antigens found on malignant T cells are also present on non-malignant T cells. This shared expression leads to the elimination of both non-malignant and malignant T cells by antibodies, resulting in immunosuppression [5,8].

The cluster of differentiation 99 (CD99) is crucial for normal T cell development [9,10], and its dysregulation in T lymphoblastic leukemia/lymphoma (T-ALL/T-LBL) can disrupt essential signaling cascades, leading to impaired differentiation, immune evasion, and, ultimately, the malignant transformation of T cells [11]. CD99 is also intensely expressed by several cancer types, particularly Ewing’s sarcoma (ES) [12,13]. Thus, CD99 has been suggested to be a promising therapeutic target molecule for an antibody drug in these CD99-overexpressing cancers. The targeting of CD99 epitopes by several antibody clones could induce cancer cell death and showed anti-cancer effects on ES, AML, and MDS in mouse xenograft models [12,13]. Recently, our in-house-produced mouse mAb MT99/3, which specifically targets the CD99 molecule, demonstrated the ability to induce apoptosis in T lymphoblastic leukemia/lymphoma (T-ALL/T-LBL) cells without causing cytotoxicity to non-malignant peripheral blood cells [14]. The mAb MT99/3 demonstrated a promising therapeutic option for T cell malignancies, with the advantage of sparing healthy cells. Additionally, aside from its anti-cancer properties, mAb MT99/3 has been proposed to regulate T cell hyperresponsiveness [15].

While the parent mAbs have shown promise in preclinical models, their translation into human therapeutics is hindered by concerns regarding immunogenicity. Humanization of antibodies addresses this issue by reducing the likelihood of eliciting an immune response in humans, while still maintaining effective interaction with human molecules. Consequently, further affinity improvement is often unnecessary when compared to phage and yeast display technologies. This study developed humanized single-chain Fv MT99/3 (HuScFvMT99/3) engineered from mouse mAb MT99/3. The construction of HuScFvMT99/3 followed the “ScFvkh” format (V-KAPPA-linker-VH). The binding activity and specificity of HuScFvMT99/3 to CD99 were explored. Additionally, the fully humanized MT99/3 (HuMT99/3) was further engineered and its potential was investigated as a targeted treatment approach for T lymphoblastic leukemia/lymphoma and hyperresponsive T cell-associated diseases.

## 2. Materials and Methods

### 2.1. Cell Lines and Cells

The T cell lines including Jurkat E6.1, MOLT-4, and SUP-T1 were purchased from American Type Culture Collection (ATCC, Manassas, VA, USA). The T cell lines and HEK293T were grown and maintained in RPMI 1640 supplemented with 10% heat-inactivated fetal bovine serum (FBS), 40 μg/mL Gentamycin, 2.5 μg/mL Amphotericin B (10% FBS-RPMI), and 10% FBS-DMEM at 37 °C in a 5% CO_2_ incubator. Peripheral blood mononuclear cells (PBMCs) were prepared using standard Ficoll–Hypaque density gradient centrifugation. PBMCs were isolated from heparinized whole blood of healthy donors or buffy coats of healthy individuals obtained from the Reginal Blood Center X, Thai Red Cross Society, Chiang Mai, Thailand.

### 2.2. Humanization and Property Validation

To achieve the humanization of the single-chain variable fragment (ScFv) for humanized antibody against human CD99 (HuScFvMT99/3), we employed the process described earlier [16]. The predicted structures of mouse ScFv anti-CD99 (MScFvMT99/3) and HuScFvMT99/3 were generated using the AI algorithm of Al-phaFold v2.3.0. The structural comparison and root-mean-square deviation (RMSD) calculation between the designed HuScFvMT99/3 and parental MScFvMT99/3 were carried out using Chimera version 1.13 software and 2StrucCompare webserver [17], respectively. The complementarity-determining regions (CDRs) of the generated HuScFvMT99/3 structure were identified using the Kabat definition in abYsis webserver [18]. The humanness score was verified using the T20 score [19]. To assess the potential immunogenicity risks associated with the grafted CDRs, the PITHA algorithm [20] was used to analyze the Al-phaFold-predicted structure of HuScFvMT99/3.

### 2.3. Construction of Plasmid-Expressing HuScFvMT99/3 (HIS6X)

The modified ScFvMT99/3 amino acid sequence was reverse-transcribed and optimized using the GenScript web service for proper expression in *E. coli*. The HuScFvMT99/3 coding sequence, containing 5’ *Nde*I and 3’ *Xho*I restriction sites, was synthesized by GenScript (Piscataway, NJ, USA). This polynucleotide was digested with *Nde*I and *Xho*I and then cloned into the *Nde*I and *Xho*I sites of the pET-21a plasmid vector to generate the pET-21a-HuScFvMT99/3 (HIS6X) plasmid.

### 2.4. Expression and Validation of HuScFvMT99/3 (HIS6X)

The pET-21a-HuScFvMT99/3 (HIS6X) plasmid was transformed into competent *E. coli* Origami B (DE3) cells to produce a humanized single-chain variable fragment of MT99/3 containing an HIS6X tag. A single colony was selected and grown in a 5 mL super broth medium starter culture overnight at 37 °C. Subsequently, the culture was inoculated into a 500 mL SB medium containing 0.05% glucose and 100 µg/mL ampicillin at 37 °C until an OD600 of 0.8 was achieved. Protein expression was induced by adding 50 µM IPTG, and this process continued for 16–18 h at 20 °C.

The induced bacteria expressing HuScFvMT99/3 (HIS6X) were washed twice with PBS and lysed via three sonication times of 5 min each at 0.5 cycles with 80% amplitude on ice. The lysed bacteria underwent freeze–thaw cycling, followed by centrifugation at 4000× *g* for 30 min at 4 °C. The cell lysate was collected and subjected to SDS-PAGE on a 15% gel with PageBlue™ (Thermo Scientific, Waltham, MA, USA) staining. Subsequently, Western blot analysis was performed to detect the HuScFvMT99/3 (HIS6X) using an optimal dilution of HRP-conjugated anti-His-tag antibody (BioLegend, San Diego, CA, USA). The reaction was developed using a chemiluminescent substrate detection system. The crude lysate containing HuScFvMT99/3 (HIS6X) was subsequently purified using Ni-NTA agarose resin (QIAGEN, Hilden, Germany).

### 2.5. Production of CD99ExhIgG and CD147Rg

CD99ExhIgG comprises the extracellular part of CD99 fused with the Fc part of human IgG. The recombinant CD99ExhIgG was produced as previously described [21]. The HEK293T cells stably expressing CD99ExhIgG were grown in 10% FBS-DMEM containing zeocin to reach around 80% confluence in a 75T flask. After that, cells were washed and cultured in a serum-free medium containing zeocin for 3 days at 37 °C in 5% CO_2_. Culture supernatants harboring CD99ExhIgG were subjected to purification by affinity chromatography using a HiTrap Protein G column. CD99ExhIgG exhibited an approximate molecular weight of 50 kDa under reducing conditions, indicative of a monomeric form. Under non-reducing conditions, it displayed an approximate molecular weight of 130 kDa, suggesting a dimeric form (Supplementary Figure S1).

CD147Rg is the extracellular part of domains 1 and 2 linked to the Fc part of human IgG. The recombinant CD147Rg was produced from CHO cells that were kindly provided by Prof. Dr. Hannes Stockinger (Medical University of Vienna, Austria). The CHO cells stably expressing CD147Rg were cultured in a serum-free medium with 5 mM methotrexate at 37 °C and 5% CO_2_. Culture supernatants containing CD147Rg were harvested and subjected to the HiTrap Protein G column for purification [22]. The molecular weight of CD147Rg was approximately 60 kDa in reducing condition (Supplementary Figure S1).

### 2.6. Validating the Ability of HuScFvMT99/3 Using Indirect ELISA

CD99ExhIgG was immobilized in the 96-well plate at 4 °C overnight. The wells were then blocked with 2% skimmed milk solution in PBS for 1 h at room temperature and then washed with 0.05% Tween 20 in PBS (washing buffer) three times. Purified HuScFvMT99/3 (HIS6X) was added to the wells at concentrations of 1, 5, and 10 μg/mL and incubated at room temperature for 1 h. After the incubation time, the plate was washed with washing buffer three times and incubated with optimal dilution of HRP-conjugated anti-His tag antibody (BioLegend) for 1 h at room temperature. The plate was then washed with washing buffer five times. 3,3′,5,5′-tetramethylbenzidine (TMB) substrate was then added to detect the enzymatic activity, and the reaction was stopped with 1N HCl. The absorbance at 450 nm was then measured using an ELISA reader. The CD147Rg-coated well was employed as a negative control.

### 2.7. Binding Inhibition Assay of HuScFvMT99/3 with Overlapping Peptide Libraries

To investigate whether HuScFvMT99/3 binds to CD99 overlapping peptides (numbers 4 to 8) recognized by mAb MT99/3 [14], the competitive ELISA was performed. Microtiter wells were coated with CD99ExhIgG at 10 μg/mL and incubated overnight at 4 °C. To block nonspecific binding, 2% skimmed milk solution in PBS was added and incubated at room temperature for 1 h. The wells were washed three times with PBS containing 0.05% Tween 20. HuScFvMT99/3 (HIS6X) at 5 μg/mL and overlapping peptides (4 to 8) at 25 μg/mL were added to the wells and incubated at room temperature for 1 h. After three additional washes with washing buffer, the wells were incubated with HRP-conjugated anti-His tag antibody (BioLegend) for 1 h. Following this incubation, the wells were washed five times and TMB substrate was added to initiate the enzymatic reaction. Absorbance at 450 nm was measured using an ELISA reader.

### 2.8. Construction of a Plasmid for HuMT99/3 Expression in HEK293T Cells

To enhance gene expression in mammalian cells, codon optimization was performed on the variable domains of HuScFvMT99/3. This optimization utilized the GenScript Codon Optimization Tool to tailor codon usage for efficient translation within mammalian systems. Synthetic DNA fragments encoding *BspE*I-*VLCL*-*Xba*I and *Not*I-*VHCH*-*Psi*I were generated and subsequently digested with their respective restriction enzymes. The purified products were then ligated into the pVITRO1-HuMT99/3-IgG1/κ plasmid, precisely replacing the original VLCL and VHCH fragments of trastuzumab [23]. This process resulted in the successful creation of the modified pVITRO1-HuMT99/3-IgG1/κ plasmid. To facilitate further experimental steps, the modified plasmid was transformed into *E. coli* XL-1 blue strain. Following transformation, several colonies were selected from LB agar plates supplemented with 100 µg/mL hygromycin B. These colonies were subjected to plasmid miniprep to isolate and purify the plasmid DNA. To confirm the accurate insertion of the codon-optimized variable domains, both restriction enzyme analysis and DNA sequencing were performed.

### 2.9. Generation of HEK293T Stably Expressing HuMT99/3

The pVITRO1-HuMT99/3-IgG1/κ plasmid was used to transfect for the expression of HuMT99/3. Poly-L-lysine was initially pre-coated, and HEK293T cells were seeded in adherence conditions in 24-well plates with DMEM supplemented with 10% FBS at 100,000 cells/well. Twenty-four hours post-seeding, 0.5 µg of pVITRO1-HuMT99/3-IgG1/κ plasmid was used to transfect the cells using Trans-IT-X2 Dynamic Delivery System (Mirus Bio, Madison, WI, USA) as transfection reagent. Twenty-four hours post-transfection, hygromycin B Gold (InvivoGen, San Diego, CA, USA) was added to the HEK293T cells at a final concentration of 25 μg/mL. The medium containing hygromycin B Gold was changed every 2 days, and cells were selected in a final concentration of 400 μg/mL.

### 2.10. Production and Purification of HuMT99/3

An HEK293T clone stably expressing HuMT99/3 was grown in 10% FBS-DMEM with 400 µg/mL of hygromycin B Gold. After the cells reached 80% confluency in 75T flask, DMEM containing 10%FBS was discarded, and cells were washed out with DMEM. After that, cells were maintained in CHO-S-SFM II media (Gibco, GrandIsland, NY, USA) containing gentamicin 40 µg/mL, fungizone 2.5 µg/mL, and hygromycin B Gold 400 µg/mL and kept at 37 °C in a 5% CO_2_ incubator for 3 days. The cultured supernatant was harvested and subjected to purification of the HuMT99/3 by using Hitrap protein G column affinity chromatography (GE Healthcare, Uppsala, Sweden). The purified HuMT99/3 was dialyzed with PBS. The concentration of HuMT99/3 was measured by the absorbance at 280 nm. The purity of HuMT99/3 was determined by using SDS-PAGE. The protein bands were developed by Coomassie brilliant blue G-250 staining.

### 2.11. Binding Activity of Purified HuMT99/3 Using Overlapping Peptide Libraries

Avidin (Sigma-Aldrich, St. Louis, MO, USA) mixed with a carbonate/bicarbonate coating buffer, pH 9.6 (10 µg/mL, 50 µL/well) was added into a 96-well ELISA plate (Corning Incorporated, Kennebunk, ME, USA) and incubated at 4 °C overnight. After washing out, the plate was blocked with 100 µL of 2% bovine serum albumin (BSA) in phosphate-buffered saline (PBS) at 37 °C for 1 h. After removing blocking agent, twelve sets of CD99 overlapping peptide libraries tagged with biotin-6-aminohexanoic acid (Ahx) at N-terminal [14] were diluted to 2 µg/mL. A volume of 50 µL of the diluted peptides was added into each well and incubated at 37 °C for 1 h. After washing out, the HuMT99/3, Dinutuximab beta (isotype-matched control Ab) at 5 µg/mL, 50 µL, or without antibodies was added into each peptide and incubated at 37 °C for 1 h. After excess antibodies were washed out, 50 µL of HRP-conjugated goat anti-human immunoglobulin Abs (Dako, Glostrup, Denmark) at dilution 1:5000 was added into each well and incubated at 37 °C for 1 h. After that, the plate was washed and TMB substrate (Invitrogen, Grand Island, NY, USA) was added. The reaction was stopped by using 1M HCl, and the absorbance was measured at 450 nm.

### 2.12. Affinity Determination of HuMT99/3 Using Bio-Layer Interferometry (BLI)

The streptavidin-coated biosensors were pre-wetted with 0.05% Tween 20 in PBS then followed by immersion in a 10 µg/mL of a CD99 peptide (Biotin-Ahx-AVVDGENDDPRPPNP) for 120 s. The excess peptide was removed by immersing the tip into 0.05% Tween 20 in PBS. Then, association step was performed and peptide-coated biosensor was incubated with a series of diluted HuMT99/3 or mouse anti-CD99 monoclonal antibody MT99/3 (mAb MT99/3) concentrations, ranging from 0.625 µg/mL to 10 µg/mL and without antibody (buffer control). After that, biosensor was dissociated using 0.05% Tween 20 in PBS buffer. This experiment was conducted using an Octet^®^ K2 BLI System (Pall ForteBio, CA, USA). The acquired data were analyzed using ForteBio data analysis software version 9.0. to determine the equilibrium dissociation constant (K**_D_**) of antibodies.

### 2.13. Binding Activity of Purified Humanized MT99/3 Using Target Cells

Immunofluorescence staining and flow cytometric analysis were used to verify the binding activity of purified HuMT99/3 against T cell lines and PBMCs. Cell lines were incubated with 10% FBS for 30 min on ice for blocking Fc receptors. The blocked cells were incubated with 10 µg/mL of HuMT99/3 or human IgG (isotype-matched control Ab) or without antibody for 30 min on ice. After unbound antibodies were washed out, bound antibodies were detected with Alexa Flour-488-anti-human IgG Abs (H+L chains specific) (Invitrogen Life Technologies, Grand Island, NY, USA) and analyzed by a BD Accuri C6 Plus flow cytometer (BD Biosciences, SanJose, CA, USA). For PBMC staining, cells were incubated with 20% human AB serum for 30 min on ice for blocking Fc receptors. The blocked cells were incubated with 10 µg/mL of biotinylated HuMT99/3 or biotinylated human IgG (isotype-matched control Ab) or without antibody for 30 min on ice. After unbound antibodies were washed out, bound antibodies were detected with FITC-conjugated streptavidin. In addition, PBMC sub-populations were simultaneously analyzed by staining with PE/Cy7-conjugated anti-CD3 mAb (BioLegend), PE-conjugated anti-CD4 mAb (BD Biosciences), and APC-conjugated anti-CD8 mAb (BD Biosciences) or PE/Cy7-conjugated anti-CD3 mAb (BioLegend) and PE-conjugated anti-CD56 mAb (BioLegend) or PE-conjugated anti-CD19 mAb (BioLegend) and PerCP-conjugated anti-CD14 mAb (BioLegend). The stained cells were analyzed by a BD Accuri C6 Plus flow cytometer. The flow cytometric data were analyzed by FlowJo software V10.6.2 (Tree Star Inc., Ashland, OR, USA).

### 2.14. Proliferation Assay

The carboxyfluorescein succinimidyl ester (CFSE) dilution technique was employed for cell proliferation assay. The human PBMC at 1 × 10^7^ cell/mL in PBS was labeled with CFSE (Sigma-Aldrich, St. Louis, MO, USA) at 1 µM at 37 °C for 10 min. The labeling was terminated by adding cold 10% FBS-RPMI. The cells were washed twice and resuspended in 10% FBS-RPMI. The CFSE-labeled cells (1 × 10^5^ cells/mL) were added into anti-CD3 mAb OKT3 (25 ng/mL)-coated well for activation. After that, 50 µL of HuMT99/3, mAb MT99/3, isotype-matched control Abs (human IgG or mouse mAb 4G2), or without Abs (medium control) were added into each well at final concentration of 20 µg/mL. Cells were cultured at 37 °C in a 5% CO_2_ incubator for 5 days. The treated cells were determined, and the reduction in CFSE for cell proliferation was determined by flow cytometry (BD Accuri™ C6). The data were analyzed by FlowJo software.

### 2.15. Cell Activation-Associated Marker Analysis

PBMCs were stimulated with anti-CD3 mAb OKT3-coated well in the presence or absence of 20 µg/mL of HuMT99/3, mAb MT99/3, or isotype-matched control Abs (human IgG or mouse mAb 4G2,) or without antibody (medium control). After that, cells were cultured at 37 °C in a 5% CO_2_ incubator. Treated cells were harvested after cultivation for 18 h then stained with FITC-conjugated anti-CD3 mAb (BD Biosciences) and PE-conjugated anti-CD69 mAb (immunotools, Friesoythe, Germany) or after cultivation for 72 h then stained with FITC-conjugated anti-CD3 mAb (BD Biosciences) and PE-conjugated anti-CD25 mAb (BioLegend). The stained cells were analyzed by flow cytometry (BD Accuri C6 Plus) and FlowJo software. By flow cytometric analysis, CD3^+^ T cells were gated and subjected to analysis for the expression of CD69 and CD25 on their surface.

### 2.16. Intracellular Cytokine Staining

PBMCs were stimulated with anti-CD3 mAb OKT3-coated well and soluble anti-CD28 mAb or kept unstimulated in the presence or absence of 20 µg/mL of HuMT99/3 and mAb MT99/3 or isotype-matched control Abs (human IgG or mouse IgG mAb 4G2) or without antibodies (medium control). For 1 h after being incubated at 37 °C in a 5% CO_2_ incubator, 1 μg/mL of brefedin A solution and 1 μM of monensin (BioLegend) were added and continuously incubated for 5 h. The treated cells were harvested and fixed with 4% paraformaldehyde in PBS at room temperature for 15 min and then permeabilized with 0.1% saponin solution (PBS containing 0.1% saponin, 5% FBS, and 0.02% NaN_3_) on ice for 15 min. Cells were stained with PE-conjugated anti-human IFN-γ, TNF-α, IL-2, or IL-17A mAbs (BioLegend) and FITC-conjugated anti-CD3 mAb (BD Biosciences) to define T cell population. The intracellular cytokines in T cells were analyzed using a flow cytometer (BD Accuri C6 Plus, BD Biosciences, Franklin Lakes, NJ, USA) and FlowJo software.

### 2.17. Cell Apoptosis Assay

T cell lines or PBMCs (1 × 10^5^/100 µL) were pre-incubated with HuMT99/3 at a final concentration of 20 µg/mL or isotype-matched control Ab (human IgG) or without antibody (medium control) in 96-well V plate for 30 min at room temperature. After incubation, the supernatants were completely discarded. Cells were then incubated with or without the secondary antibody crosslinker goat anti-human IgG antibody (Jackson ImmunoResearch Laboratories, West Grove, PA, USA) at 20 µg/mL, 150 µL for 30 min at room temperature. After that, the treated cells were incubated at 37 °C in a 5% CO_2_ incubator for 0, 90, and 180 min. For mouse mAb MT99/3, cells were pre-incubated with mAb MT99/3 at a final concentration of 20 µg/mL. Then, culture supernatants were discarded and 150 µL of the secondary antibody crosslinker goat anti-mouse IgG antibody (Jackson ImmunoResearch Laboratories) at 5 µg/mL was added. The mAb MT99/3-treated cells were then incubated at 37 °C for 0, 90, and 180 min. Treated cells were harvested and stained with apoptosis markers Annexin V-FITC and 7-AAD solution (BioLegend). Cell apoptosis was determined by a BD Accuri C6 Plus flow cytometer (BD Biosciences) and analyzed by using FlowJo software.

### 2.18. Thermal Stability Testing of HuMT99/3 and mAb MT99/3

To evaluate the impact of the framework substitution, we assessed the thermal stability of HuMT99/3 and mAb MT99/3. The predicted structures of HuMT99/3 and mAb MT99/3 were submitted for analysis of their thermal stability using the ScooP tool [24].

### 2.19. Statistical Analysis

All statistical analyses were performed using GraphPad Prism version 9.3.1 (GraphPad Software, Boston, MA, USA). Data were expressed as mean ± SD. The two-way ANOVA and unpaired *t*-test were used for comparison. *p* < 0.05 was considered statistically significant.

## 3. Results

### 3.1. Suitable Frameworks for Generating HuScFvMT99/3

**Figure 1:**
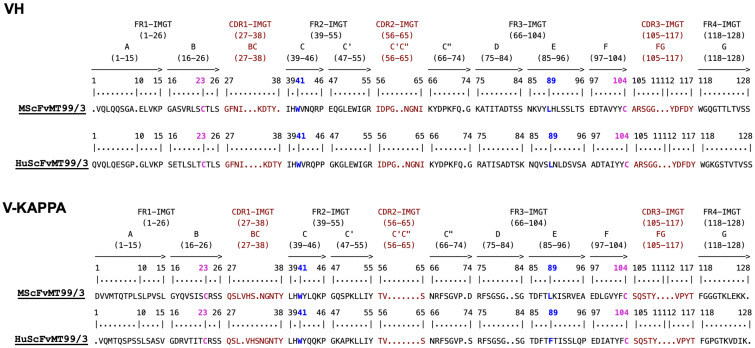
Amino acid sequences of the VH and V-KAPPA domains for MScFvMT99/3 and HuScFvMT99/3. Framework regions (FRs) and CDR delimitations and gaps are delineated according to IMGT numbering. Conserved position 23 (C23) and 104 (C104) are indicated as magenta. Conserved position 41 (W41) and hydrophobic 89 are indicated as blue. The percentage identity of *Homo sapiens* genes is as follows: IGHV1-3*01 (62.9%) and IGHJ4*01 (85.7%) for VH-MScFvMT99/3 and IGHV4-4*08 (70.4%) and IGHJ4*01 (78.6%) for VH-HuScFvMT99/3. For the V-KAPPA domains, the percentage identity is IGKV2D-29*02 (80%) and IGKJ2*01 (83.3%) for V-KAPPA-MScFvMT99/3 and IGKV1-33*01 (77.8%) and IGKJ3*01 (91.7%) for V-KAPPA-HuScFvMT99/3. The lengths of CDR-IMGT are [8.8.10] and [11.3.9] for VH and V-KAPPA, respectively. This alignment was created using IMGT/DomainGapAlign [25].

**Figure 2:**
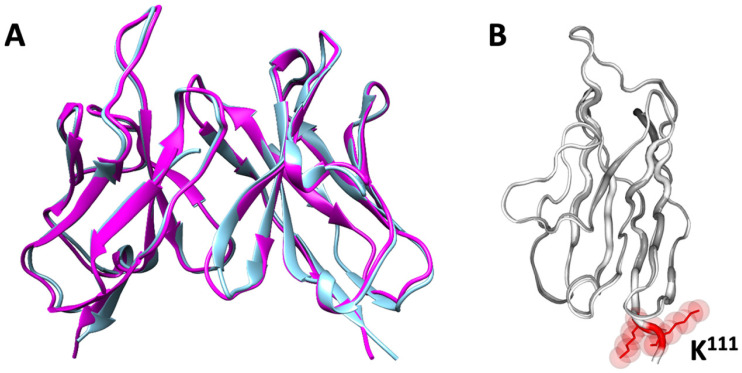
Comparative structural analysis of MScFvMT99/3 and HuScFvMT99/3 generated by AlphaFold. (**A**) The conformational structure of MScFvMT99/3 (blue ribbon) and HuScFvMT99/3 (magenta ribbon) was compared using Chimera version 1.13 software. Analysis on the 2StrucCompare server showed RMSD of 0.98 Å for the VH and 0.85 Å for the VL. (**B**) The coordinate of K^111^ of VL domain within HuScFvMT99/3 structure, which influences the RMSD is depicted.

The mouse (3JAU) and human (5UQY) variable domain templates were selected based on their highest structural similarity and sequence homology, as determined using Bioluminate (Schrödinger software, version 2020-3). The 3JAU crystal structure was selected as a template for predicting the conformational structure regarding the amino acid sequences of MScFvMT99/3 deduced from hybridoma cDNA sequencing. Subsequently, human frameworks from 5UQY were retrieved to replace the frameworks of the modeled MScFvMT99/3 structure, ensuring the preservation of the dimension of antibody CDR loops. The humanness scores of the HuMT99/3 V_H_ domain increased from 67.53% to 80.75%, while the V_L_ domain improved from 85.56% to 94.75%. These substantial increases represent a significant step toward minimizing immunogenicity and enhancing the suitability for human therapy. Nevertheless, examination of HuMT99/3 through PITHA analysis revealed that its immunogenicity remains relatively high for therapeutic applications. Regarding the IMGT numbering system, the framework regions (FRs) and CDRs of the VH and V-KAPPA domains for MScFvMT99/3 and HuScFvMT99/3 were demonstrated (Figure 1). The comparative model of MScFvMT99/3 and HuScFvMT99/3 generated by AlphaFold is shown in Figure 2A. RMSD analysis using 2StrucCompare demonstrated a high degree of structural similarity between MScFvMT99/3 and HuScFvMT99/3, with values of 0.96 Å for the V_L_ domain and 0.85 Å for the V_H_ domain. Notably, while the V_L_ domain exhibited slightly lower similarity than the V_H_ domain, the observed difference at residue K^111^ is not located within the antigen-binding site, suggesting its potential for limited impact on antigen recognition (Figure 2B).

### 3.2. Observation of HuScFvMT99/3 Expression in E. coli Origami B (DE3)

**Figure 3:**
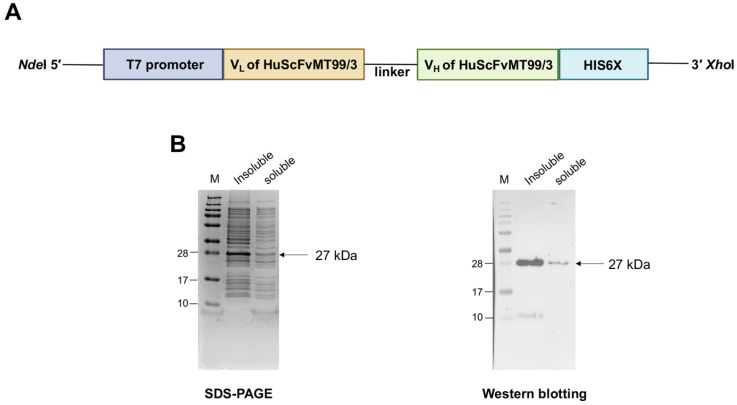
Schematic of plasmid construction and expression of HuScFvMT99/3 in bacterial system. (**A**) Plasmid composition of pET-21a-harboring HuScFvMT99/3, flanked by an HIS6X tag at the C-terminus. (**B**) Crude HuScFvMT99/3 was subjected to SDS-PAGE stained with PageBlue™ and Western blotting under reducing condition. The membrane was probed with HRP-conjugated anti-His-tag mAb, and the protein bands were visualized using chemiluminescent substrate. M; Molecular weight markers in kDa. Original images of (**B**) can be found in Supplementary Materials.

pET-21a plasmid-harboring HuScFvMT99/3 coding sequences was constructed (Figure 3A) and proven for the correct insertion by DNA sequencing. The expression of HuScFvMT99/3 (HIS6X) in *E. coli* Origami B (DE3) crude extract was verified using SDS-PAGE and Western blot analysis. A distinct protein band at 27 kDa was observed in insoluble and soluble fractions, corresponding to the expected size of HuScFvMT99/3 with the HIS6X tag (Figure 3B).

### 3.3. Binding of HuScFvMT99/3 (HIS6X) to CD99 Antigenic Determinant

**Figure 4:**
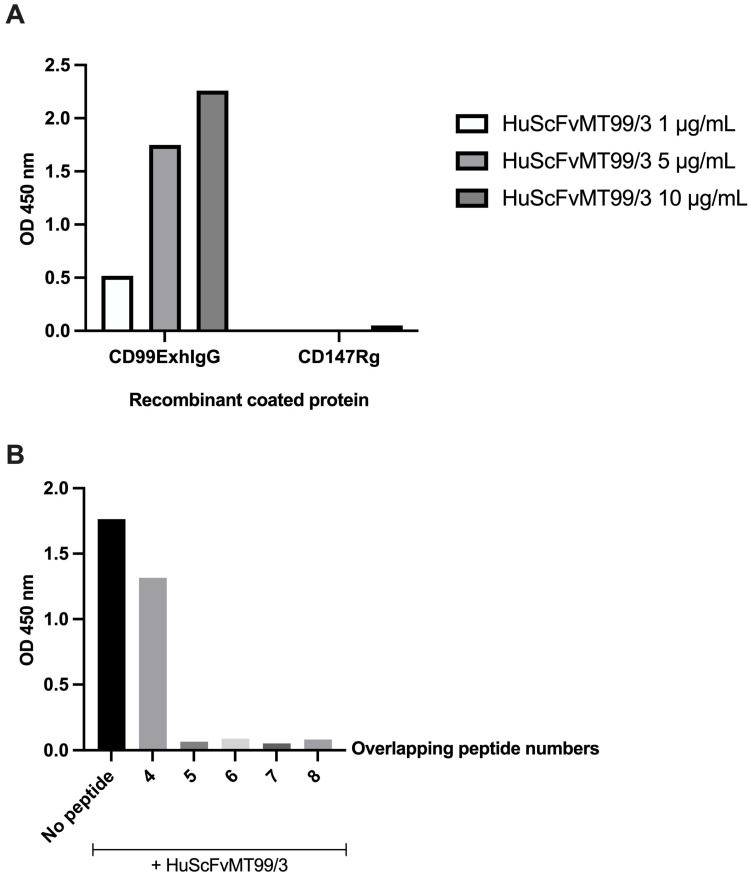
Binding activity and specificity of HuScFvMT99/3 against CD99. (**A**) The microtiter wells were immobilized with CD99ExIgG, and purified HuScFvMT99/3 (HIS6X) was subsequently added at various concentrations. CD147Rg-coated well was employed as negative control. The binding activity of HuScFvMT99/3 (HIS6X) was detected by HRP-conjugated anti-His-tag mAb. (**B**) CD99 overlapping peptides 4 to 8 were used to compete with HuScFvMT99/3 binding to CD99ExhIgG-coated wells. HRP-conjugated anti-His-tag mAb was used to monitor the binding activity. After adding the substrate, the optical density at 450 nm of these experiments was measured by an ELISA reader.

The capacity of HuScFvMT99/3 (HIS6X) to bind to CD99ExhIgG was validated using an indirect ELISA approach. The findings consistently demonstrate a robust interaction between the HuScFvMT99/3 (HIS6X) construct and CD99ExhIgG, affirming the retention of its binding functionality (Figure 4A). As the concentration of HuScFvMT99/3 (HIS6X) increased, absorbance values exhibited a corresponding rise in a dose-dependent concentration manner, and no interaction was observed between HuScFvMT99/3 and CD147Rg. Furthermore, the binding of HuScFvMT99/3 toward CD99ExhIgG was inhibited using candidate peptides of human CD99 numbers 4 to 8 as depicted in Figure 4B. These data indicate the antigenic determinant of CD99 recognized by HuScFvMT99/3.

### 3.4. Purity of HuMT99/3 Prepared from HEK293T

**Figure 5:**
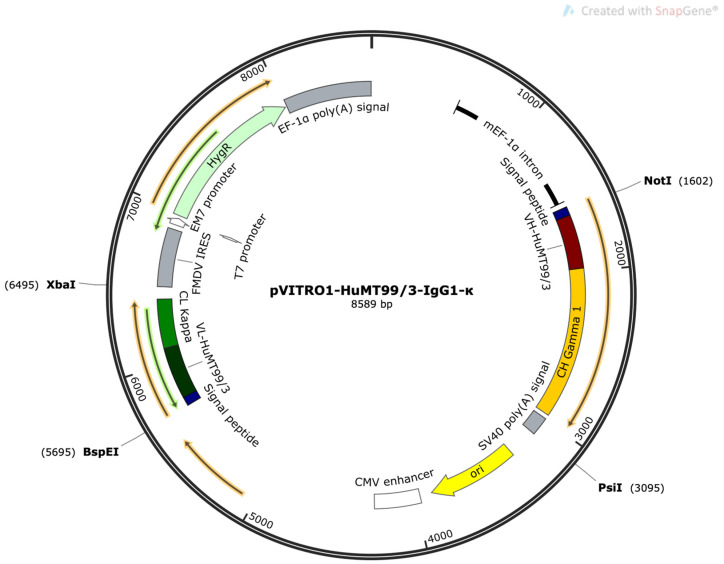
The constructed pVITRO1-HuMT99/3-IgG1/κ plasmid. The plasmid vector was created with SnapGene^®^ Viewer 6.1.2 software. Gene encoding for the variable heavy chain of HuMT99/3 (VH-HuMT99/3), the constant heavy chain of human IgG1 (CH Gamma 1), the variable light chain of HuMT99/3 (VL-HuMT99/3), and the constant kappa light chain (CL Kappa) are illustrated. Homo sapiens IGHG1*01 (G1m17,1 CH1 K120, CH3 D12, and L14) [26] is used for CH Gamma 1.

**Figure 6:**
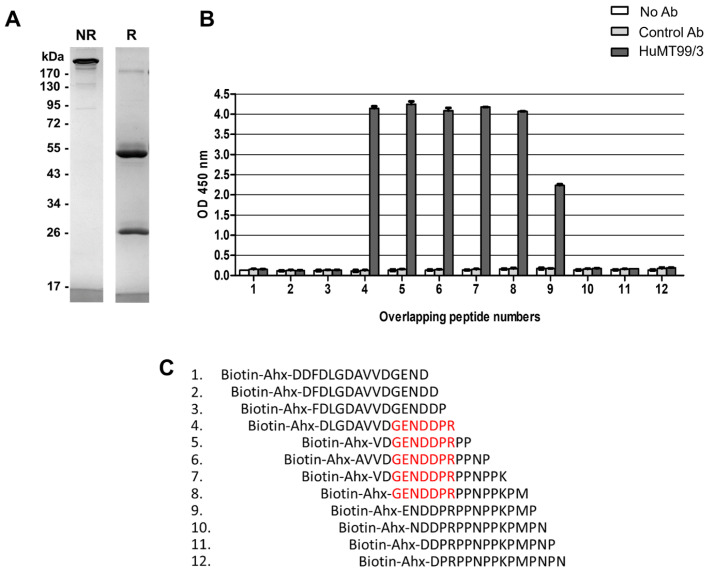
Purity and epitope recognized by HuMT99/3. (**A**) Purified HuMT99/3 was subjected to SDS-PAGE under non-reducing condition and reducing condition (R). The protein bands were developed by Coomassie brilliant blue G-250 staining. Molecular weight markers in kDa are indicated on the left. Original images of (**A**) can be found in Supplementary Materials. (**B**) Binding activity of HuMT99/3 to twelve sets of CD99 overlapping peptide libraries. The bar graphs show mean ± SD of two independent experiments. (**C**) Amino acid sequences of 12 overlapping peptides are shown. The predicted amino acid residues of CD99 epitope recognized by HuMT99/3 are indicated in red letters.

The constructed pVITRO1-HuMT99/3-IgG1/κ plasmid (Figure 5) was transfected into HEK293T, and the stable clones were generated using 400 μg/mL hygromycin B. The transfected HEK293T cells were further maintained in a selective medium and purified using the protein G column. The purity of HuMT99/3 was determined by SDS-PAGE. In a non-reducing condition, a major band at a molecular weight above 170 kDa was observed. The result demonstrates a high purity of harvested HuMT99/3. In reducing conditions, two major bands of HuMT99/3 at approximately 50 kDa and 25 kDa, which were related to heavy chains and light chains of antibody isotype IgG, were observed (Figure 6A). These results indicate that humanized anti-CD99 mAb was successfully produced as a full IgG structure.

### 3.5. Epitope Recognition of HuMT99/3 and mAb MT99/3 on CD99

According to the identification of the CD99 epitope recognized by mouse mAb MT99/3 using twelve sets of CD99 overlapping peptide libraries [14], we therefore verified whether the HuMT99/3 would bind to the similar epitope of mAb MT99/3. The high purity of mouse mAb MT99/3 as shown in Supplementary Figure S2 was used. By the same CD99 overlapping peptide libraries, the HuMT99/3 showed strong positive reactivity with peptide numbers 4 to 8 and weak positive reactivity with peptide number 9 (Figure 6B). Consistent with mAb MT99/3, HuMT99/3 bound the GENDDPR epitope within the CD99 molecule (Figure 6C) [14].

### 3.6. The Binding Properties of HuMT99/3 and mAb MT99/3

**Figure 7:**
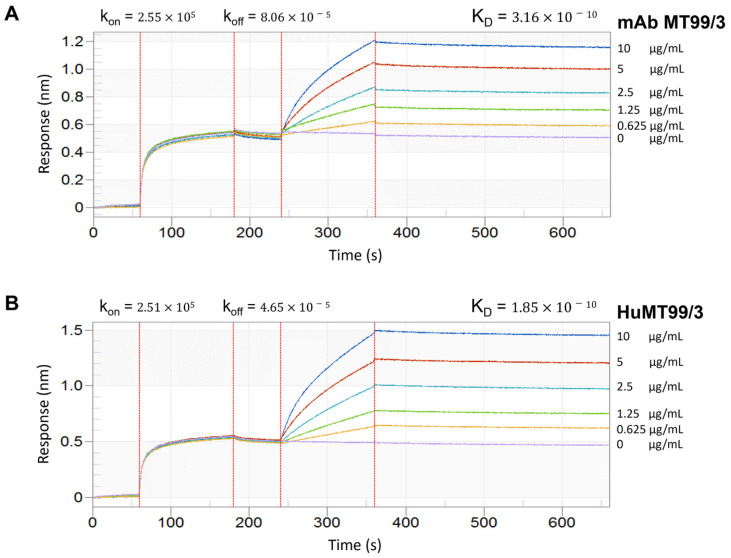
The binding affinity of HuMT99/3 compared with mouse MT99/3. Bio-layer interferometry (BLI) was performed. The CD99 peptide (Biotin-Ahx-AVVDGENDDPRPPNP) at 10 μg/mL was loaded onto streptavidin biosensor followed by PBST buffer (60 s to 240 s). After that, mAb MT99/3 (**A**) or HuMT99/3 (**B**) was loaded at the indicated concentrations for the association step followed by PBST buffer for the dissociation step (240 s to 660 s). The binding affinities K_D_ (M), k_on_ (1/Ms), and k_off_ (1/s) are indicated.

**Figure 8:**
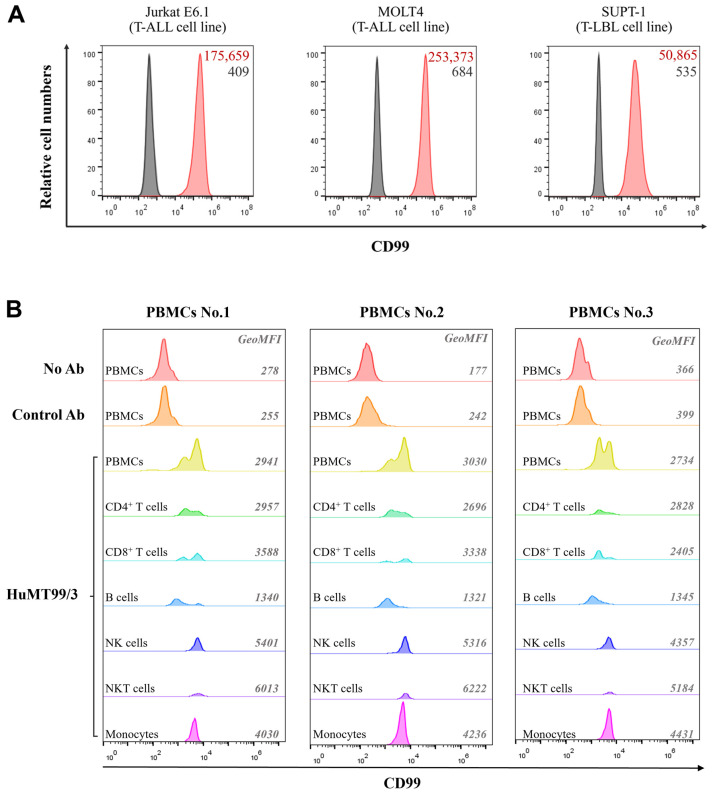
The binding activity of HuMT99/3 against CD99 on target cells. (**A**) Three T cell lines and (**B**) human PBMCs from three healthy individuals were used to perform indirect immunofluorescence staining. For cell lines, cells were stained with HuMT99/3 (red peaks), human IgG as an isotype-matched control Ab (clear peaks), or without mAbs (gray peaks) followed by Alexa Flour-488-conjugated anti-human IgG Abs. The histogram graphs exhibit CD99 expression levels. Geometric mean fluorescence intensity of CD99 expression (red color) and isotype-matched control Ab staining (black color) are indicated at right upper corner of each histogram graph. For PBMCs, cells were stained with biotinylated HuMT99/3, biotinylated human IgG as an isotype-matched control Ab (Control Ab), or without mAbs (No Ab) followed by FITC-conjugated streptavidin. Sup-populations of PBMCs were gated for analysis of CD99 expression including CD4^+^ T cells (CD3^+^CD4^+^), CD8^+^ T cells (CD3^+^CD4^+^), B cells (CD19^+^), NK cells (CD3^−^CD56^+^), NK T cells (CD3^+^CD56^+^), and monocytes (CD14^+^). The histogram graphs exhibit CD99 expression levels on PBMC sub-populations. Geometric mean fluorescence intensity (GeoMFI) of CD99 expression is indicated at right of each histogram graph.

The binding affinity of HuMT99/3 and mAb MT99/3 was then compared by bio-layer interferometry (BLI). As shown in Figure 7, the HuMT99/3 (KD = 1.85 × 10^−10^ M) exhibits binding affinity comparable to that of the mouse-derived MT99/3 (KD = 3.16 × 10^−10^ M). Moreover, the binding activity of HuMT99/3 against the full-length CD99 was confirmed using CD99-expressing myeloma cells [27]. The flow cytometry experiment showed that HuMT99/3 binds specifically to CD99-expressing myeloma cells. There is no background signal observed in non-expressing myeloma cells. Additionally, CD99 peptide number 6 containing a CD99 epitope of the HuMT99/3 could complete the binding activity of the HuMT99/3 on CD99-expressing myeloma cells (Supplementary Figure S3). As CD99 expression levels were determined in malignant T-ALL cell lines including Jurkat E6.1 and MOLT-4 and a T-LBL cell line SUP-T1, as well as human PBMCs using mAb MT99/3 [14], in this study, CD99 expression on these cells was confirmed by indirect immunofluorescence staining using HuMT99/3. The HuMT99/3, but not the isotype-matched control Ab (human IgG), showed positive reactivity with all tested cells (Figure 8). Among malignant T cells, HuMT99/3 strongly reacted to T-ALL cells (Figure 8A). For normal human PBMCs, HuMT99/3 showed positive reactivity with all sub-populations at different expression levels (Figure 8B). These results indicate the binding reactivity of the HuMT99/3 specifically against the human CD99 native form.

Additionally, the thermal stability of HuMT99/3 and mAb MT99/3 was analyzed using the ScooP tool. The results indicate that both structures exhibit comparable melting temperatures (Tm), standard folding enthalpies (ΔHm), and standard folding heat capacities (ΔCp) (Supplementary Table S1). Therefore, the selected frameworks are suitable for humanization.

### 3.7. Inhibitory Effects of HuMT99/3 on T Cell Activation

**Figure 9:**
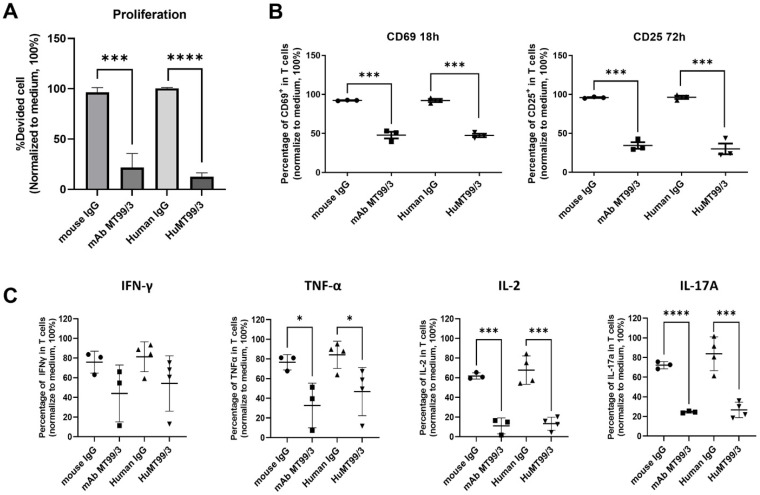
Inhibitory effects of HuMT99/3 compared with mAb MT99/3 on T cell activation. Upon anti-CD3 mAb OKT3 activation, PBMCs were treated with or without 20 µg/mL of mAb MT99/3, HuMT99/3, or isotype-matched control Abs (mouse IgG or Human IgG). (**A**) CFSE dilution technique was used for cell proliferation analysis after activation for 5 days. (**B**) T cell activation-associated markers CD69 and CD25 expressions in CD3^+^ T cells were determined by direct immunofluorescence staining at 18 and 72 h, respectively. (**C**) Cytokine production in CD3^+^ T cells was determined by intracellular staining. All experiments were analyzed by flow cytometry. Each data point in the presence of the indicated antibody was normalized relative to its medium control as 100%. The bar graphs or scatter plots exhibit mean ± SD. Unpaired *t*-test was used for comparison; * *p* < 0.05, *** *p* < 0.001, and **** *p* < 0.0001.

According to our previous report, mAb MT99/3 could inhibit proliferation, T cell activation-associated markers, and Th1 and Th17 cytokine production upon activation of peripheral blood mononuclear cells with the anti-CD3 mAb [15]. In this study, the inhibitory effects of HuMT99/3 were compared with mAb MT99/3 upon T cell activation. The results show that HuMT99/3 had an inhibitory effect the same as mAb MT99/3. The HuMT99/3 and mAb MT99/3 significantly inhibited anti-CD3 mAb OKT3-activated PBMC proliferation (Figure 9A). These anti-CD99 antibodies dramatically suppressed CD69 and CD25 expressions on T cells upon T cell activation (Figure 9B). Furthermore, HuMT99/3 and mAb MT99/3 could significantly reduce TNF-α, IL-2, and IL-17A and tend to reduce IFN-γ production in T cells upon T cell activation (Figure 9C). These results indicate that HuMT99/3 was successfully generated and was able to keep the functional activities of mAb MT99/3.

### 3.8. Direct Killing Effect of HuMT99/3 on Malignant T Cells

**Figure 10:**
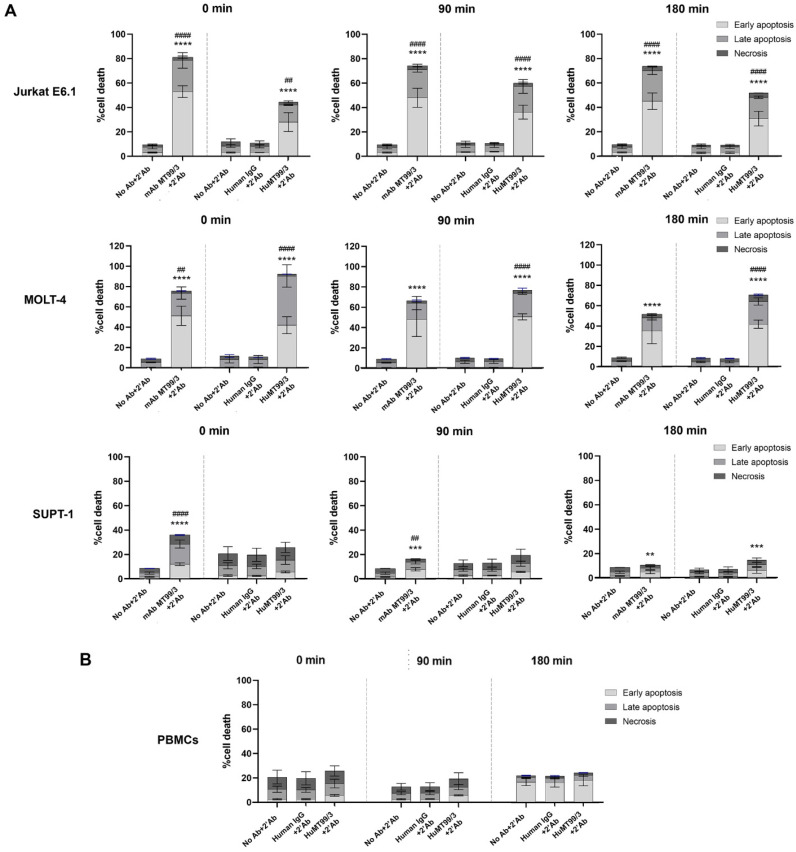
Direct killing effect of HuMT99/3 on malignant T cells and healthy PBMCs. (**A**) malignant T cells and (**B**) healthy PBMCs were treated with 20 µg/mL of HuMT99/3, Human IgG (isotype-matched control Ab), or no Ab (medium control) in the presence of secondary antibody crosslinker at 0, 90, and 180 min. mAb MT99/3 with secondary antibody crosslinker was used as positive control for inducing apoptosis in malignant T cells. The treated cells were stained with Annexin-V-FITC and 7-AAD. The percentages of cell death in early apoptosis (Annexin V^+^7-AAD^−^), late apoptosis (Annexin V^+^7-AAD^+^), and necrosis (Annexin V^−^7-AAD^+^) were analyzed by flow cytometry. The bar graphs express mean ± SD of three independent experiments. Two-way ANOVA followed by Tukey’s test was used for early apoptosis induced by HuMT99/3 vs. human IgG or mAb MT99/3 vs. No Ab; ** *p* < 0.01, *** *p* < 0.001, and **** *p* < 0.0001; and for late apoptosis induced by HuMT99/3 vs. human IgG or mAb MT99/3 vs. No Ab; ^##^ *p* < 0.01 and ^####^ *p* < 0.0001.

Our previous study demonstrated that mAb MT99/3 induced apoptosis in T-ALL/T-LBL but not in healthy PBMCs in the presence of a secondary antibody crosslinker [14]. In this study, the direct killing effect of HuMT99/3 was investigated on T-ALL/T-LBL cells and healthy PBMCs. Jurkat E6.1 and MOLT-4 were representative of T-ALL cells. SUPT-1 was a representative of T-LBL cells. Upon HuMT99/3 treatment with a secondary antibody crosslinker, apoptosis of T-ALL/T-LBL was induced by HuMT99/3 like the mAb MT99/3 positive control (Figure 10). Most importantly here, the HuMT99/3 confirmed that it did not induce apoptosis of healthy PBMCs corresponding to our previous report [14]. The results indicate that HuMT99/3 might be a promising antibody for the killing of T-ALL/T-LBL with low cytotoxicity to normal cells.

## 4. Discussion

Human CD99 is a type I transmembrane glycoprotein which is ubiquitously expressed on various cell types [28,29]. CD99 exhibited higher expression levels in T cell acute lymphoblastic leukemia (T-ALL) than normal T cells by about seven times [30]. The CD99 molecule is a multifunctional protein. It acts as an oncogenic function in various cancer cell types, including T cell lineage leukemia/lymphoma [28]. Thus, CD99 was used as a marker for detecting minimal residual disease (MRD) of T-ALL and was proposed to be a promising target for antibody treatment of T cell malignancy [30,31,32]. In our previous study, we demonstrated that mouse mAb MT99/3 binds a new CD99 epitope at amino acid VDGENDDPRPP residues 60–70 [14]. The mAb MT99/3 induced T-ALL/T-LBL apoptosis but not normal peripheral blood cells [14]. Consequently, the mAb MT99/3 might be a promising antibody for the treatment of T-ALL/T-LBL with efficacy and fewer adverse events. In addition to malignant T cells, CD99 plays a crucial role in T cell regulation [33,34]. According to our previous study, targeting CD99 by mouse mAb MT99/3 reduced the ability of antigen presentation on monocytes leading to hypofunction of T cells. Upon OKT3-activated PBMC, T cell functions including T cell proliferation, T cell marker expression, and Th1 and Th17 cytokine production were suppressed by mAb MT99/3 [15]. Therefore, the mAb MT99/3 was proposed to be used for the treatment of hyperresponsive T cell-associated conditions. To develop mouse mAb MT99/3 for clinical use, a humanized antibody derived from mouse mAb MT99/3 named HuMT99/3 was generated in this study.

Based on the structural validation of HuScFvMT99/3, the RMSD of VH and VL closely resembles the predicted structure of MScFvMT99/3. The preserved immunoreactivity of HuScFvMT99/3 expressed in *E. coli* Origami B (DE3) was confirmed by ELISA. Moreover, the specific binding epitope recognition of HuScFvMT99/3 after CDR grafting was also observed using biotinylated human CD99 peptides. These findings confirm that the humanization process did not impair the binding activity and specificity. Interestingly, the inhibition analysis revealed that peptide number 4 exhibited a minimal inhibitory effect on the binding between HuScFvMT99/3 and CD99ExhIgG, compared to peptides 5 to 8. This phenomenon supports the aforementioned report on the specific binding epitope of anti-CD99 mAb with VDGENDDPRPP residues [14]. Accordingly, the residues P69P70 are crucial for the recognition of HuScFvMT99/3.

The HuMT99/3 with the IgG1 isotype was successfully engineered and produced in HEK293T cells. The humanization process utilizing CDR grafting onto a selected human framework might lead to a significant loss of binding affinity for the specific antigen [35]. Consequently, the binding affinity of HuMT99/3 against the specific epitope peptide number 6 was validated in comparison to the mAb MT99/3. The binding affinity and specificity against CD99 are comparable to those reported previously for mouse mAb MT99/3 [14], suggesting that CDR grafting does not adversely affect the binding affinity. Additionally, the demonstration of a similar amino acid sequence defining the epitope of mAb MT99/3 was elucidated using overlapping peptide libraries. While HuScFvMT99/3 exhibits a demonstrably enhanced humanness T20 score, PITHA analysis indicates high predicted immunogenicity for HuScFvMT99/3, requiring further optimization. Despite PITHA predicting high immunogenicity, the Z-scores of the hydrophobic surface (1.5) and total charge (0.8) of the CDR region in HuMT99/3 fall within the modest range observed among FDA-approved therapeutic antibodies. Leveraging the human origin of the chosen frameworks, abYsis can be employed for targeted CDR engineering to refine specific humanness aspects [18]. However, the prediction of specific determining regions (SDRs) is necessary but hindered by the unavailability of a CD99 conformational structure.

The purified HuMT99/3 could recognize native CD99 expressed on malignant T cells and normal PBMCs. Furthermore, HuMT99/3 retains its ability to inhibit T cell activation and proliferation, while also attenuating T cell activation markers and the production of Th cytokines [15]. Interestingly, HuMT99/3 demonstrates efficacy in inducing apoptosis in T-ALL/T-LBL cells, as evidenced by previous studies while exhibiting no toxicity to normal PBMCs [14]. For clinical use, humanized antibodies offer advantages over mouse antibodies. Humanized antibodies have reduced immunogenicity, minimizing the generation of antibodies against mouse immunoglobulins in patients. Additionally, the Fc region of humanized antibodies is suitable for activating human effector functions, aiding in the eradication of cancer cells [3,36]. The success achieved in generating humanized antibodies against human molecules signifies a promising avenue for accelerating the development of therapeutic antibodies. However, further research is imperative to elucidate the molecular mechanisms underlying the disparate outcomes observed in normal cells.

## 5. Conclusions

This study successfully humanized the mouse monoclonal antibody MT99/3, which targets CD99, a glycoprotein overexpressed in T-ALL. The humanized antibody, HuScFvMT99/3, was developed through CDR grafting and retained the specificity and binding affinity of the original antibody. The superimposed structures of MScFvMT99/3 and HuScFvMT99/3 generated by AlphaFold indicate a high degree of structural similarity. HuMT99/3, produced in HEK293T cells, maintained its ability to bind CD99 on malignant T cells, while effectively inhibiting T cell activation, proliferation, and cytokine production. Similar to MT99/3, the humanized version induced apoptosis in T-ALL cells without affecting PBMCs. Despite predictions of high immunogenicity, HuMT99/3 exhibited biophysical properties within acceptable ranges for FDA-approved antibodies, suggesting that further refinement could lower immunogenicity risk. HuMT99/3 shows significant therapeutic potential for T-ALL with reduced immunogenicity and maintained efficacy, though further research is required to optimize its immunogenicity and better understand its impact on both malignant and normal cells.

## Data Availability

The original contributions presented in the study are included in the article/Supplementary Materials, and further inquiries can be directed to the corresponding authors.

## Supplementary Materials

The following supporting information can be downloaded at https://www.mdpi.com/article/10.3390/biom14111422/s1, Figure S1: SDS-PAGE analysis of purified CD99ExhIgG and CD147Rg; Figure S2: SDS-PAGE analysis of purified mAb MT99/3; Figure S3: Binding activity of HuMT99/3 on myeloma and CD99-expressing myeloma cell; Table S1: Thermal stability analysis of HuMT99/3 and mAb MT99/3. The original files of SDS-PAGE and Western blot can be found in Supplementary Material Files.
