# Supplementary material for: Exploring the Biological Activity of a Humanized Anti-CD99 ScFv and Antibody for Targeting T Cell Malignancies
Source: Biomolecules. 2024 Nov 8;14(11):1422. doi: 10.3390/biom14111422 (PMC11592157; doi:10.3390/biom14111422)

Supplementary materials

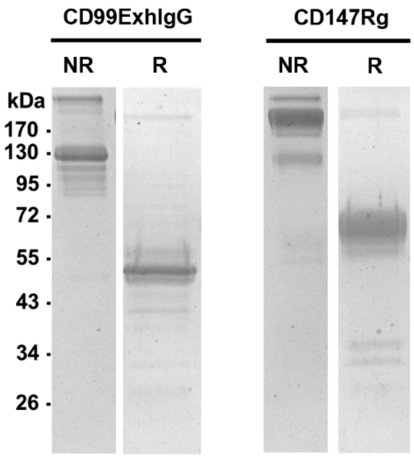

Supplementary Figure S1. Purified CD99ExhIgG and CD147Rg were subjected for SDS-PAGE under non-reducing condition (NR) and reducing condition (R). The protein bands were developed by Coomassie brilliant blue G-250 staining. Molecular weight markers in kDa were indicated on the left.

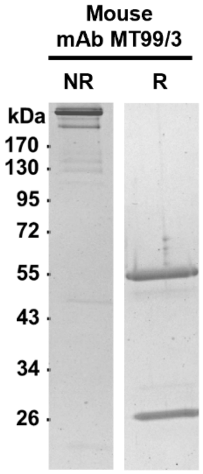

Supplementary Figure S2. Purified mouse mAb MT99/3 were subjected for SDS-PAGE under non-reducing condition (NR) and reducing condition (R). The protein bands were developed by Coomassie brilliant blue G-250 staining. Molecular weight markers in kDa were indicated on the left.

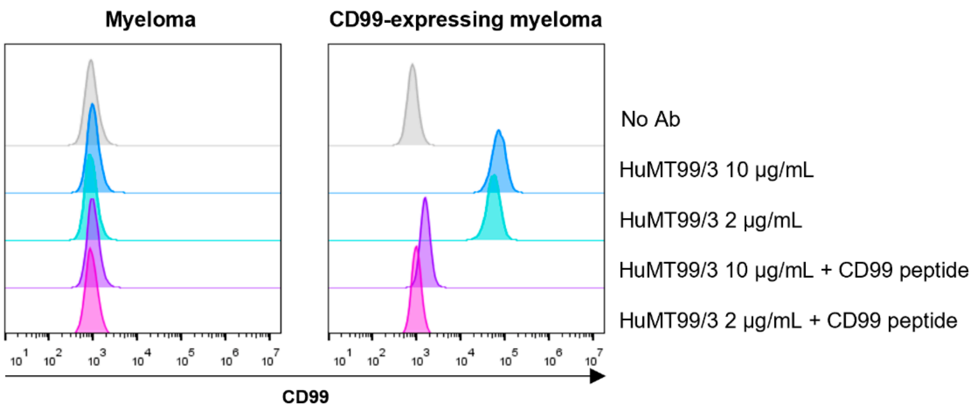

Supplementary Figure S3. Binding reactivity of humanized MT99/3**.** Myeloma cells and human CD99-expressing myeloma cells were stained with 10 µg/mL or 2 µg/mL of HuMT99/3 in the presence or absence of a 50 µg/mL of a CD99 peptide (Biotin-Ahx-AVVDGENDDPRPPNP) or without antibody (No Ab). The bound antibodies were detected with Alexa Flour-488-anti-human IgG Abs and analyzed by flow cytometry.

Supplementary Table S1. Thermal stability analysis comparing HuMT99/3 and mAb MT99/3 was conducted using ScooP.

**Antibody**	**Chain**	***Tm*** (°C)	**Δ*Hm*** (kcal/mol)	**Δ*Cp*** (kcal/mol K)
HuMT99/3	H-chain	62.4	-94.7	-1.84
mAb MT99/3		63.0	-91.6	-1.78
HuMT99/3	L-chain	63.1	-96.2	-1.75
mAb MT99/3		64.2	-96.3	-1.97

Original gel/blot images

Uncropped gel/blot of Figure 3B

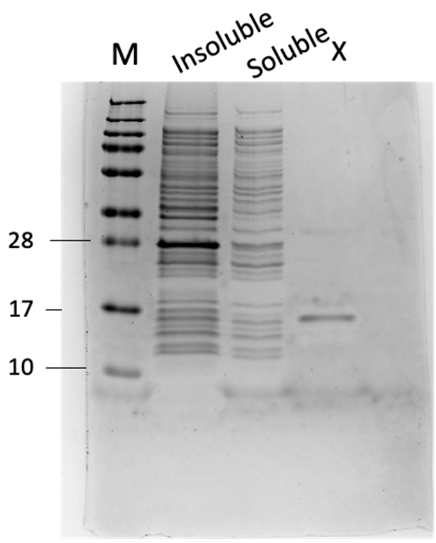

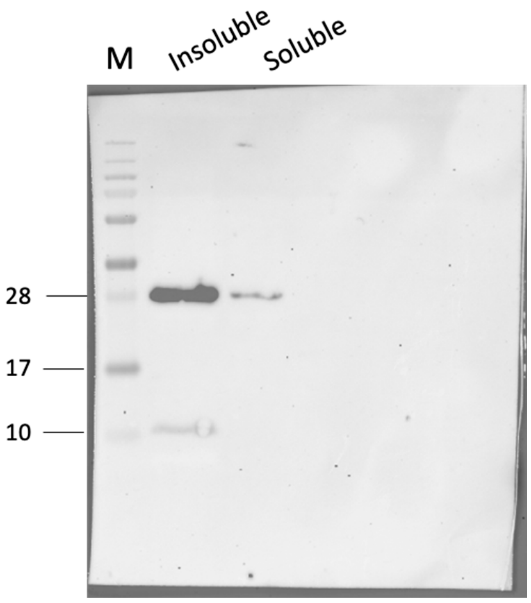

Uncropped gel of Figure 6A

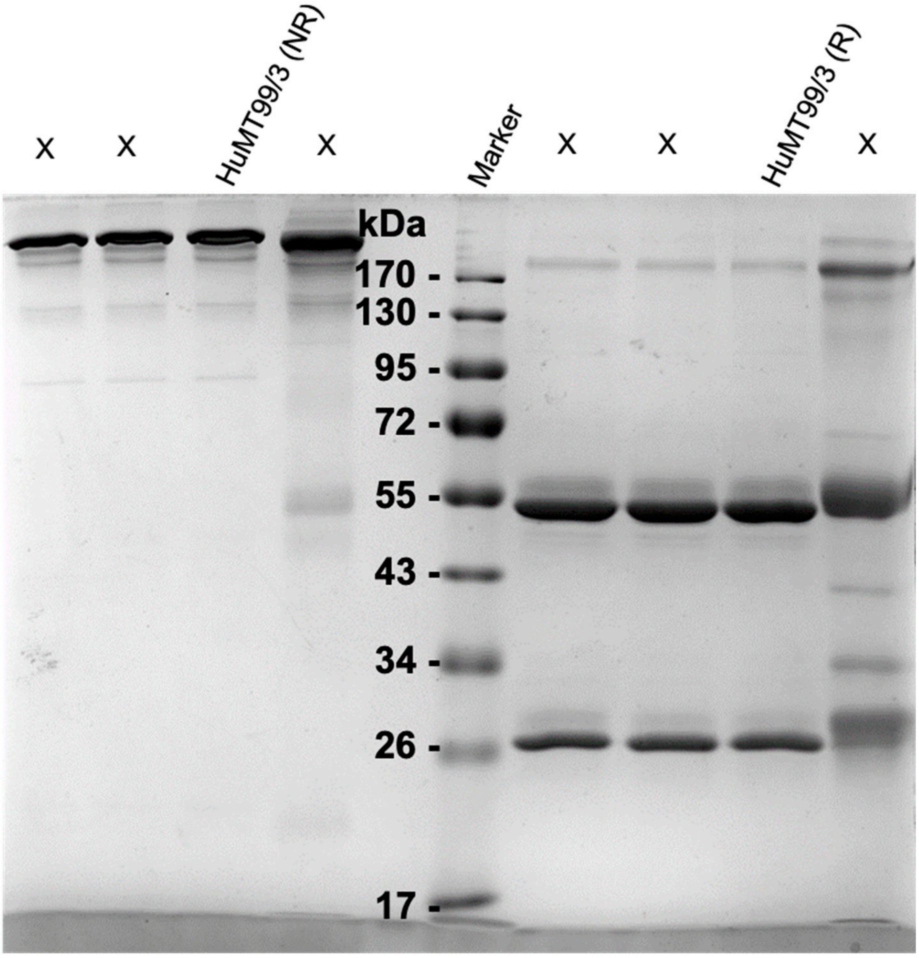

Uncropped gel of Figure S1 and S2

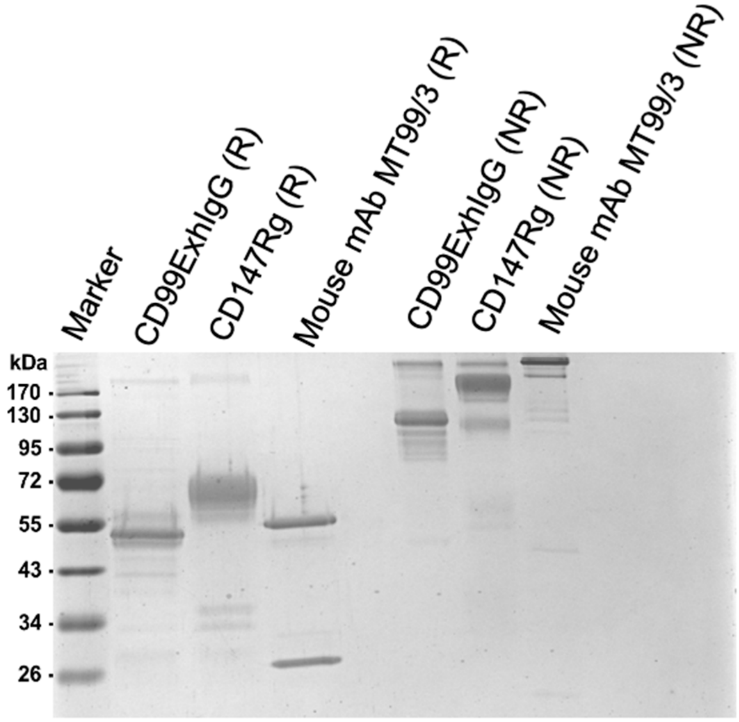

Supplement: Supplementary file 1 [file biomolecules-14-01422-s001.zip › Supplementary materials_Biomolecules_Final.pdf]
