# Supplementary material for: Exploring the Biological Activity of a Humanized Anti-CD99 ScFv and Antibody for Targeting T Cell Malignancies
Source: Biomolecules. 2024 Nov 8;14(11):1422. doi: 10.3390/biom14111422 (PMC11592157; doi:10.3390/biom14111422)
Supplement: Supplementary file 1 [file biomolecules-14-01422-s001.zip › Supplementary Table S1.pdf]

Supplementary Table S1. Thermal stability analysis comparing HuMT99/3 and mAb MT99/3 was conducted using ScooP.

**Antibody**	**Chain**	***Tm*** (°C)	**Δ*Hm*** (kcal/mol)	**Δ*Cp*** (kcal/mol K)
HuMT99/3	H-chain	62.4	-94.7	-1.84
mAb MT99/3		63.0	-91.6	-1.78
HuMT99/3	L-chain	63.1	-96.2	-1.75
mAb MT99/3		64.2	-96.3	-1.97
