# Supplementary figures and images for: Exploring the Biological Activity of a Humanized Anti-CD99 ScFv and Antibody for Targeting T Cell Malignancies
Source: Biomolecules. 2024 Nov 8;14(11):1422. doi: 10.3390/biom14111422 (PMC11592157; doi:10.3390/biom14111422)

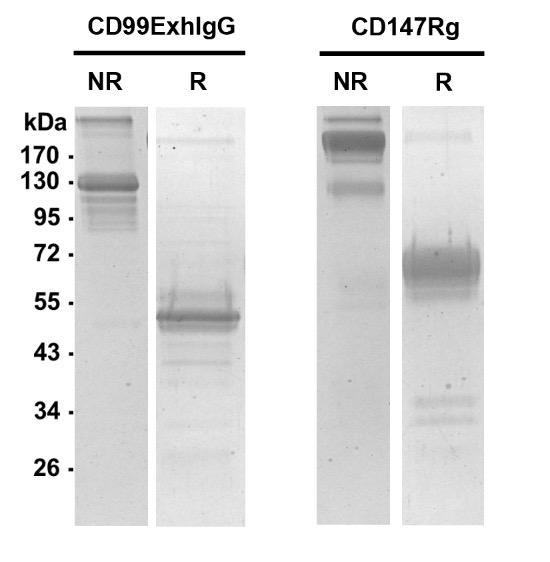

Supplement: Supplementary file 1 [file biomolecules-14-01422-s001.zip › Figure S1.jpg]

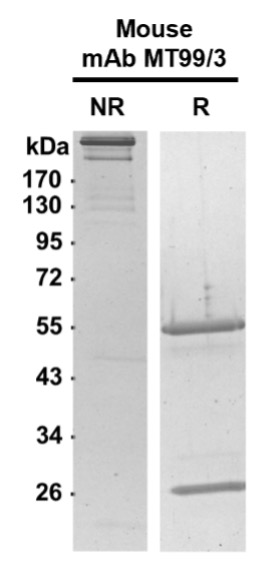

Supplement: Supplementary file 1 [file biomolecules-14-01422-s001.zip › Figure S2.jpg]

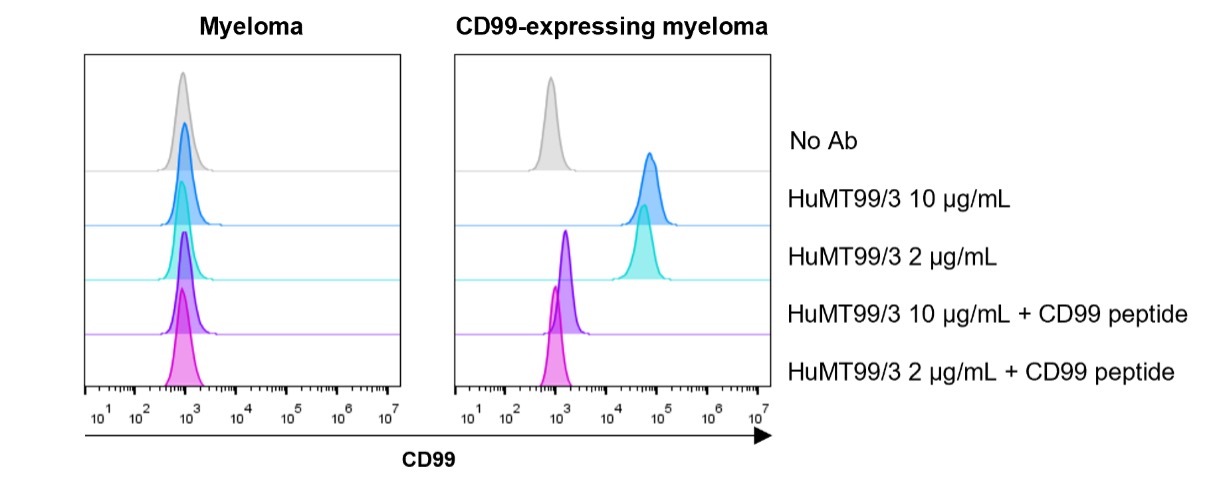

Supplement: Supplementary file 1 [file biomolecules-14-01422-s001.zip › Figure S3.jpg]

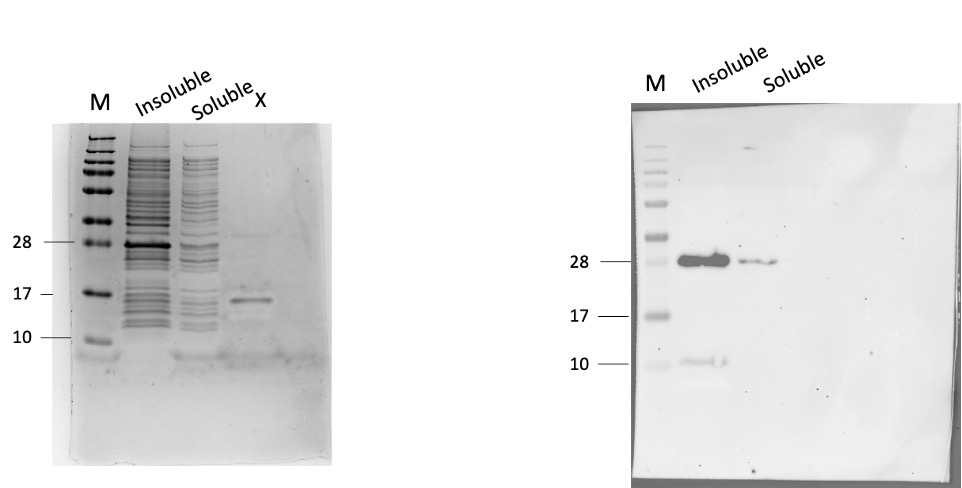

Supplement: Supplementary file 1 [file biomolecules-14-01422-s001.zip › Uncroped gel_blot Figure 3B.jpg]

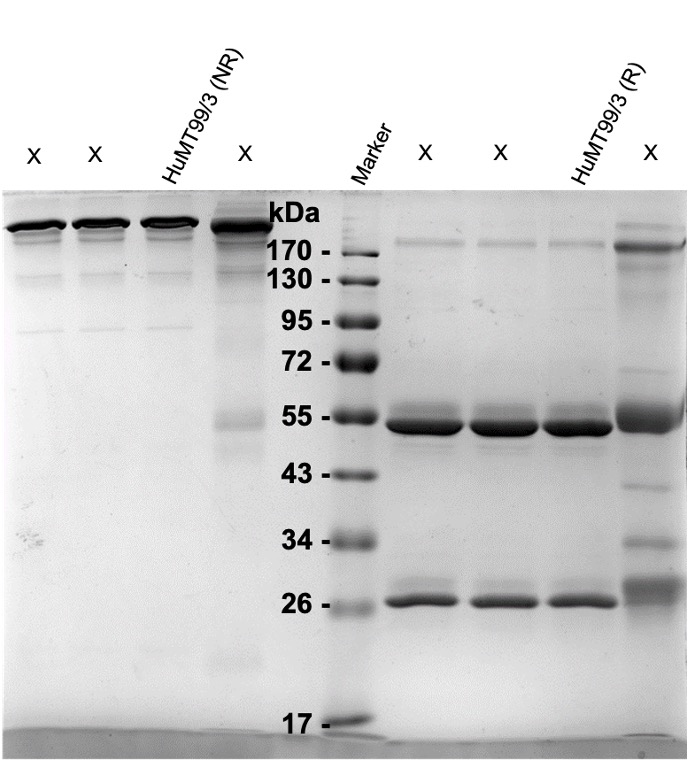

Supplement: Supplementary file 1 [file biomolecules-14-01422-s001.zip › Uncroped gel_blot Figure 6A.jpg]

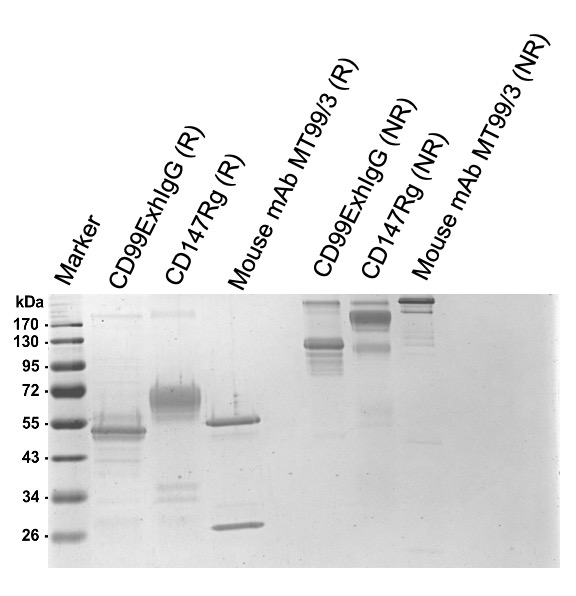

Supplement: Supplementary file 1 [file biomolecules-14-01422-s001.zip › Uncroped gel_blot Figure S1 and S2.jpg]
